# Loop diuretic use in patients with AKI: different severity, different response

**DOI:** 10.1186/s13054-018-2097-7

**Published:** 2018-08-19

**Authors:** Yanfei Shen, Muying Wu

**Affiliations:** 1 Intensive care unit, Dongyang People’s Hospital, NO.60 Wuning West Road, Jinhua City, Zhejiang 322100 People’s Republic of China; 2 Intensive care unit, Yiwu Tianxiang East Hospital, NO.188 Huancheng South Road, Yiwu City, Zhejiang People’s Republic of China

Recently, Dr. Bove and his colleagues performed an excellent systematic review [1] and reported that furosemide exhibited a neutral effect in acute kidney injury (AKI) treatment (odds ratio (OR) 1.14; 95% CI 0.75 to 1.72). Despite being well designed, several limitations should be noted. First, the potential heterogeneity needs to be addressed. In this meta-analysis, three different interventions were combined in the control group, including placebo, continuous furosemide infusion, and torasemide administration. However, from a clinical aspect, comparability of therapeutic strategies is a prerequisite for a meta-analysis. Simply taking all these interventions as one control treatment is inappropriate and difficult to interpret for clinical meaning, despite the heterogeneity not being significant (I^2^ = 0). Second, the effect of furosemide in AKI remains inconsistent. We noticed that the associations between poor outcomes and furosemide were more frequently reported in cohorts with higher serum creatinine (sCr) (3.8 mg/dl [2], 3.3 mg/dl [3]) while insignificant in patients with mild AKI (1.8 mg/dl [4]). Considering that fluid accumulation is a common issue in AKI, we speculate that patients with mild AKI are more likely to respond to furosemide challenge and the side effects of furosemide, such as oxidative stress [5], may be overwhelmed by the reduced fluid accumulation. In the current comparison, we performed a subgroup analysis according to different control treatments (Additional file 1). In all four studies using placebo as control (Cantarovich [6], Cantarovich [7], Kleinknecht [8], and Shilliday [9]), all the patients were described as having acute kidney failure (which indicates severe AKI) and the pooled effect was insignificant (OR 0.93; 95% CI 0.54 to 1.59; *p* = 0.78). However, in another four studies using continuous furosemide infusion as control treatment, only one study (Brown [10]) was reported as ARF, and an extremely high dose of furosemide was used in this study (1000 mg/24 h, bolus vs 3000 mg/24 h, continuous). The severity of AKI in another three studies (sCr 1.4 mg/dl in Kunt [11], 2.1 mg/dl in Schuller [12], and 1.3 mg/dl in Shah [13]) was mild and when excluding these studies, the pooled outcome showed that continuous furosemide infusion was associated with lower mortality (OR 3.82; 95% CI 1.30 to 11.28; *p* = 0.024). Thus, we think combining different treatment strategies as control treatment may cause biased conclusions. Finally, this is an enlightening study, and further investigations regarding whether continuous furosemide therapy could reduce the mortality rate in AKI patients are needed.

## Additional file

Additional file 1: **Figure S1.** Subgroup meta-analysis according to different interventions of the control group. (TIF 776 kb)

## Abbreviations

AKI: Acute kidney injury
ARF: Acute renal failure
OR: Odds ratio
sCr: Serum creatinine
